# Molecular cloning, characterization and expression analysis of Frizzled 6 in the small intestine of pigs (*Sus scrofa*)

**DOI:** 10.1371/journal.pone.0179421

**Published:** 2017-06-14

**Authors:** Lijun Zou, Xiaocheng Wang, Liping Jiang, Shengping Wang, Xia Xiong, Huansheng Yang, Wei Gao, Min Gong, Chien-An A. Hu, Yulong Yin

**Affiliations:** 1 Key Laboratory for Agro-Ecological Processes in Subtropical Regions, Institute of Subtropical Agriculture, the Chinese Academy of Sciences, Changsha, Hunan, China; 2 Laboratory of Animal Nutrition and Human Health, College of Life Sciences, Hunan Normal University, Changsha, Hunan, China; 3 Laboratory of Basic Biology, Hunan First Normal College, Changsha, Hunan, China; 4 School of Basic Medical Science, Central South University, Changsha, Hunan, China; 5 Jiangxi Science & Technology Normal University, Nanchang, Jiangxi, China; Western University, CANADA

## Abstract

*Frizzled 6* (*FZD6*) encodes an integral membrane protein that functions in multiple signal transduction pathways, for example, as a receptor in Wnt/planar cell polarity (PCP) signaling pathway for polarized cell migration and organ morphogenesis. Mutations in *FZD6* have been identified in a variety of tumors. In this study, the full-length cDNA of *Sus scrofa FZD6* (*Sfz6*) was cloned and characterized. Nucleotide sequence analysis demonstrates that the *Sfz6* gene encodes the 712 amino-acid (aa) protein with seven transmembrane domain. Tissue distribution analysis showed that *Sfz6* mRNA is ubiquitously expressed in various tissues, being highest in kidney, moderate in jejunum, ileum, colon, liver, and spleen. However, FZD6 protein is highly expressed in the heart and there was no significant difference in other tissues. The relative abundance and localization of FZD6 protein in jejunum along the crypt-villus axis was determined by Western blot and immunohistochemical localization. The results show that in the jejunum, FZD6 protein is highly expressed in the villus and less in the crypt cells. Cellular proliferation and viability assays indicate that knockdown of *FZD6* with small interfering RNAs (siRNA) significantly reduced the cell viability of the intestinal porcine enterocyte cells (IPEC-J2). Furthermore, qPCR and Western blot analysis revealed that expressions of ras-related C3 botulinum toxin substrate 1 (*Rac1*); ras homolog gene family member A (*RhoA*) and c-Jun N-terminal kinase 1 (*JNK1)*, some components of PCP signaling pathway were upregulated (*P* < 0.05) by knockdown of *FZD6* in IPEC-J2 cells. In conclusion, these results showed that FZD6 abundance in the villus was higher than that in crypt cells and knockdown of *FZD6* induces PCP signal pathway components expression in IPEC-J2 cells. Our findings provide the foundation for further investigation into porcine *FZD6* gene.

## Introduction

Wnts, a family of evolutionarily conserved, cysteine-rich, secreted glycoproteins, functions as central mediators of vertebrate and invertebrate development, influencing cell proliferation, differentiation, and migration [1, 2]. In mammals, the *Wnt* gene family consists of at least 19 members encoding secreted glycoproteins functioning as ligands for receptors [3, 4]. Wnts bind cell surface Frizzled receptors and activate at least one of the three distinct signaling pathways, which include β-catenin-dependent Wnt/β-catenin pathway, β-catenin-independent Wnt/planar cell polarity (PCP) and Wnt/calcium pathway[5]. Frizzled (FZD), a family of seven transmembrane domain proteins, have extracellular cysteine-rich domain (CRD) at the amino terminus that have been implicated as the Wnt binding domain [5]. Previous studies have classified FZD as G-protein-coupling receptors (GPCR) [6, 7]. In addition to FZD, the Wnt/β-catenin pathway requires the low-density lipoprotein receptor related proteins 5 and 6 (Lrp5/6) as co-receptor [8].

Ten members of the FZD receptor family (FZD 1–10) have been identified in mammals. FZD6 is the largest protein in the FZD family[9]. FZD6 mainly transduces PCP signaling, serving as a mediator of polarized cell movement (cell migration) and organ morphogenesis[10], as well as of cytoskeletal pathways, such as the small GTPases RhoA and cdc42, Rho kinase, protein kinase C (PKC) and JNK1[9–11]. Miyakoshi *et al* showed that Wnt-4 can activate the β-catenin-dependent Wnt pathway and binds the *FZD6* CRD in kidney epithelial cells [12]. More interesting to us is that the β-catenin-dependent Wnt signaling cascade plays a crucial role in driving the proliferation of the intestinal epithelial cells and *FZD6* has previously been detected in the crypt and differentiated epithelial cells of the mouse small intestine and colon [13]. In addition, *FZD6* was highly expressed in intestine mucosal layer of adult human patients with ulcerative colitis (UC) and Crohn's disease, however, the precise function of *FZD6* in the small intestine is not clear[14].

At present, the *Homo sapiens FZD6* (*Hfz6*), *Mus musculus FZD6* (*Mfz6)* and *Bovine FZD6* (*Bfz6*) have been cloned. However, the sequence and molecular mechanism of *Sfz6* remains unknown. In this study, we set out to study the role of *Sfz6*, cloned and characterized a full-length *Sfz6* cDNA and investigated expression of *FZD6* at both mRNA and protein levels in various tissues and along the crypt-villus axis of the jejunum. In addition, we also analyzed the effects of *FZD6* knockdown on proliferation and apoptosis in IPEC-J2 cells.

## Material and methods

### Animal breeding and sample collection

For this study, six 5-month-old pigs (Duroc × Landrace × Yorkshire) were purchased from the Hunan Ground Biological Science and Technology Co., Ltd. (Changsha, China). All pigs were sacrificed by jugular puncture under general anesthesia via intravenous injection of 4% sodium pentobarbital solution (40 mg/kg BW), and then immediately eviscerated [15]. The small intestines were separated and cleaned several times with ice-cold phosphate-buffered saline (PBS). Samples of the jejunum, ileum, colon, liver, heart, pancreas, spleen, and kidney were collected from each animal, immediately frozen in liquid nitrogen, and stored at −70C until subsequent analysis.

Additional jejunal segments were collected for epithelial cells isolation and immunohistochemistry analyses. The sequential isolation of pig small intestinal epithelial cells along the crypt-villus axis was based on the method of Fan *et al*, which was developed to quantify the specific activities of several major digestion- and absorption-related enzymes in differentiated enterocytes [16]. Briefly, the divided mid-jejunum segments were rinsed thoroughly with ice-cold physiological saline solution and incubated at 37°C for 30 min with oxygenated PBS. The fluid contents of the intestinal segments were drained and discarded. The jejunum segments were then filled with 15–30 mL isolation buffer (5 mM Na_2_EDTA, 10 mM HEPES pH 7.4, 0.5 mM dithiothreitol [DTT], 0.25% bovine serum albumin [BSA], 2.5 mM D-glucose, 2.5 mM L-glutamine and 0.5 mM DL -β-hydroxybutyrate sodium salt, oxygenated with an O_2_/CO_2_ mixture [19:1, v/v]) and incubated for 20 or 30 min in a shaking water bath incubator at 37°C. After incubation, the contents of the jejunum segment, which included buffer and isolated cells, were transferred to a 250 mL conical centrifuge bottle. This procedure was repeated six times to yield six “cell fractions” (F1 to F6). Each of the first three cell fractions (F1 to F3) was collected after separate 10 min incubations (total incubation time, 30 min), whereas each of the last three fractions (F4 to F6) was collected after separate 20 min incubations (total incubation time, 30 min). The cell fractions were washed twice as follows: each cell fraction was centrifuged at 400 × *g* for 4 min at 4°C, the supernatant was discarded, and the cell pellet was dispersed in 15–30 mL oxygenated cell resuspension buffer (10 mM HEPES, 1.5 mM CaCl_2_, and 2.0 mM MgCl_2_, pH 7.4). The cells were immediately frozen at −80°C for enzyme marker assays and western blot analysis.

The experimental design and procedures used in this study were carried out in accordance with the Chinese Guidelines for Animal Welfare and Experimental Protocols, and approved by the Animal Care and Use Committee of the Institute of Subtropical Agriculture at the Chinese Academy of Sciences. All institutional and national guidelines for the care and use of laboratory animals were followed; and all efforts were made to minimize animal suffering.

### RNA extraction and cDNA synthesis

Total RNA was extracted from the samples using TRIzol^®^ Reagent (Invitrogen-Life Technologies, Carlsbad, CA, USA) following the manufacturer’s protocol, and was dissolved in DEPC-treated water. The quality and concentration of the extracted RNA were checked by 1.2% agarose gel electrophoresis and spectrophotometry using a NanoDrop^®^ ND2000 (NanoDrop Technologies Inc., Wilmington, DE, USA). Then, 1.0 μg total sample RNA was incubated with DNase I (Fermentas, St Leon-Rot, Germany) and reverse-transcribed using the Reverse Transcription System (Promega Corporation, Madison, WI, USA). Finally, the cDNAs were stored at −80°C until further processing [17].

### Cloning of the *Sfz6* gene

Primers to recognize the *Sfz6* cDNA sequence were designed with Primer 5.0 version (PREMIER Biosoft International, Palo Alto, CA) based on the conserved nucleotide acid sequences of the mouse and human *FZD6* cDNA sequences. The full-length cDNA of *Sfz6* was amplified using mixed cDNA from several tissues (jejunum, ileum, colon, and liver) as a template. The primers used for cloning were—5′—CTC CTG AGG TGG CTG AAA T—3′ as the forward primer and 5′—GAG GGT GGT ATG TGG TTG TC—3′ as the reverse primer. The PCR mixture (25 μL total volume) contained 12.5 μL 2X Taq PCR master mix (Tiangen, Beijing, China), 8.5 μL ddH_2_O, 2 μL mixed cDNA, and l μL (10 nM) each primer. The PCR reaction was performed using an Eppendorf Mastercycler (Hamburg, Germany) with the following parameters: initial denaturation at 94°C for 5 min; 35 cycles of denaturation at 94°C for 60 s; annealing at 55°C for 60 s, and elongation at 72°C for 60 s; and a final elongation at 72°C for 10 min. PCR products (4 μL) were identified by electrophoresis at 120 V for 50 min on a 1% (m/v) agarose gel. The amplification products were extracted from the gel using the Promega Wizard SV Gel and PCR Clean-up Systems, and the purified PCR products were cloned into the PMD18TM vector (TaKaRa, Dalian, China) and the resultant constructs were sequenced at Invitrogen (Guangzhou, China).

### Quantitative real-time PCR (qPCR) analysis

The expression profile of mRNA was determined by real-time PCR analysis. Primers were designed with Primer 5.0 based on the gene sequence of pig to produce an amplification product (Table 1). GAPDH was used as a housekeeping gene to normalize target gene transcript levels. Quantitative PCR was performed on the ABI PRISM 7900 HT platform (Applied Biosystems, Inc., Foster City, CA, USA) using SYBR^®^ Premix Ex TaqTM II (TaKaRa) following the suggested protocol. The results were analyzed by ABI 7900 SDS software (version 2.3).

**Table 1:** Primers used for real-time PCR analysis.

Gene	Primer sequences (5'-3')	Size,bp	Tm,°C
*FZD6*	F: GAAGGATAAGAGCCGAGTGC	159	59
	R: TGAACAAGCAGTGATGTGGAG		
*RhoA*	F: GATGAGCACACAAGGCGTGA	114	61
	R: TGCTGAACACTCCATGTACC		
*Rac1*	F: ACCATTGTCCCAACACTCC	162	62
	R: GGCTTCGTCGAACACTGTCT		
*JNK1*	F: CGCTACTACAGAGCACCTGAG	116	57
	R: ACCTGGGAACAAAACACCAC		
*GAPDH*	F: ATGGTGAAGGTCGGAGTGAA	154	61
	R: CGTGGGTGGAATCATACTGG		

### Bio-informatics analysis

BLAST was used to identify homologous sequences in the GenBank databases. Sequences were aligned in the multiple alignment program CLUSTAL V [18]. The neighbor-joining method was used to construct the phylogenetic tree. The transmembrane domain of the protein was predicted using a Transmembrane Hidden Markov Model (Version 2.0) [19].

### Enzyme activity and western blot analysis

Alkaline phosphatase activities in isolated cell fractions were determined using an enzyme assay kit, according to the manufacturer’s protocol (Nanjing Jiancheng Bioengineering Institute, Nanjing, China). Western blot analysis was performed as described by Xiong *et al*.[20]. Ice-cold RIPA lyses buffer (Biyuntian Biotech Co., Ltd., Shanghai, China) containing 0.1 mM phenylmethylsulfonyl fluoride (PMSF) was used to extract the total protein fraction from the tissue and cells. The sample was then centrifuged, and the protein concentration of the resulting supernatant was assessed prior to western blot detection of target proteins. The following Primary antibodies used were as follow: Santa Cruz: N-terminal extracellular domain of FZD6 (1:500; goat polyclonal, sc-32148), RhoA (1:3000; mouse monoclonal, sc-418), Rac1 (1:3000; rabbit polyclonal, sc-217); Abcam: pan-cadherin (1:1000; rabbit monoclonal, ab51034), JNK (1:2000; mouse monoclonal, ab201624), GAPDH (1:5000; mouse monoclonal, ab8245).

### Immuno-histochemical localization

Immunohistochemistry (IHC) was performed as previously described [21]. Jejunal segments from 5-month-old pigs were fixed overnight in 10% neutral buffered formalin and embedded in paraffin. After dewaxing and hydration, 5 μm sections were incubated in methanol with 3% H_2_O_2_ for 30 min at room temperature and then treated for antigen retrieval by boiling in citrate antigen retrieval solution for 30 min. The sections were blocked with 5% BSA in PBS and incubated with primary antibodies (anti-Frizzled 6: 1:50 dilution) overnight at 4°C. After washing with 0.1 M PBST, pH 7.4 for 5 min each, thrice. Antibodies were detected using the Polink-2 plus Polymer HRP detection system for goat primary antibodies (PV-9003, ZSGB-Bio, Beijing, China) according to the manufacturer’s instructions. The reaction was visualized with a 3-3′-diaminobenzidine tetrahydrochloride (DAB) kit (ZLI-9017, ZSGB-Bio). Then, the sections were counterstained with Harris hematoxylin, differentiated using hydrochloric acid ethanol (75% alcohol and 1% hydrochloric acid), and blued using water. After dehydration in a graded alcohol series (70%, 85%, 95% and 100%) and clearing with dimethylbenzene, the slides were mounted with neutral gum. Densitometry was quantified with the Image-Pro Plus analytical imaging system (Media Cybernetics, Bethesda, MD, USA) as described previously [22]. Briefly, 6 digital images at 400 fold magnifications were captured by the CH-9435 CCD camera (Leica, Switzerland) coupled to a Leica DM 3000 microscope. A total of five fields selected from hot-spot areas were acquired in villus or crypt region per slide. Integrated optical density (IOD) of all the positive staining in each field and area of interest (AOI) was measured. The IOD was used to evaluate the area and intensity of the positive staining. The mean density (IOD/AOI) represented the concentration of specific protein per unit area.

### Cell culture and siRNA transfection

IPEC-J2 Cells were cultured in DMEM supplemented with 10% FBS, 100 units/mL penicillin, and 100 ug/ml streptomycin and incubated in a humidified atmosphere at 37°C with 5% CO_2_. Using the small interfering RNA (siRNA) designer (Invitrogen, Inc.), three siRNA sequences of *sfz6* gene were found (Table 2). The siRNA or negative control were transfected using Lipo2000 (Thermo, USA) by the standard protocol. Cells only transfected with Lipo2000 were used as blank group. After 48 hours, transfected cells were collected and processed for qPCR, Western blot.

**Table 2:** The small interfering RNA sequences of porcine *FZD6* gene.

siRNA-*FZD6*-1	Sense strand	5'-GCAAUAGUACAGCCUGCAATT-3'
	Antisense strand	5'-UUGCAGGCUGUACUAUUGCTT-3'
siRNA-*FZD6*-2	Sense strand	5'-GCUGGCAUUAUUUCCUUAATT-3'
	Antisense strand	5'-UUAAGGAAAUAAUGCCAGCTT-3'
siRNA-*FZD6*-3	Sense strand	5'-CCAUUGUCGUCAGUACCAUTT-3'
	Antisense strand	5'-AUGGUACUGACGACAAUGGTT-3'
Negative Control (N.C)	Sense strand	5'-UUCUCCGAACGUGUCACGUTT-3'
	Antisense strand	5'-ACGUGACACGUUCGGAGAATT-3'

### Cell proliferation assay

Cell proliferation was assessed by CCK-8 (Dojindo, Japan). Briefly, 6 × 10^3^ cells were seeded in each 96-well plate for 24 h and then transfected with *FZD6* siRNA (30nm, 50nm and 100nm) using Lipo2000 incubated for 24, 48 and 72 hours, respectively. CCK-8 reagent was added to each well at 1 hour before the endpoint of incubation. The absorbance at 450 nm of each well was recorded by microplate reader (Bio Tek, USA).

### Annexin V/ Propidium Iodide (PI) staining assay

Further apoptotic effects of *FZD6* against IPEC-J2 cells after 48 h was examined using an Annexin V FITC Apoptosis Detection Kit I (BD Pharminge^™^, USA). 1×10^6^ cells were treated with 30nm, 50nm and 100 nm *FZD6* siRNA-1 for 48 hours. Then, the cells were collected by centrifuging at 400 × *g* for 3 minutes. The supernatant was discarded, and the cell pellet was washed twice with cold PBS and then suspended in 100 μl of binding buffer. Next, the cells were incubated for 15 minutes at room temperature (25°C) in the dark with a mixture of 5 μl of FITC Annexin V and 5 μl PI. Then, 400 μl of binding buffer were loaded, and the analysis was completed using a BD FACSCanto^™^ II Flow Cytometer (BD Bioscience, San Jose, CA, USA). Untreated cells were considered a negative control. Cells were gated to exclude cell debris, cell doublets, and cell clumps.

### Statistical analysis

All results of are presented as the means ± SEM. The data of DAB signal quantified were compared using Student’s t-test. The other data were analyzed by SPSS 19.0 statistical package (SPSS, Chicago, IL, USA). Data for multiple comparisons were subjected to one-way ANOVA followed by Duncan test and a value of *P* < 0.05 was considered statistically significant.

## Results

### *Sfz6* cDNA cloning and homology analysis

**Fig 1:**
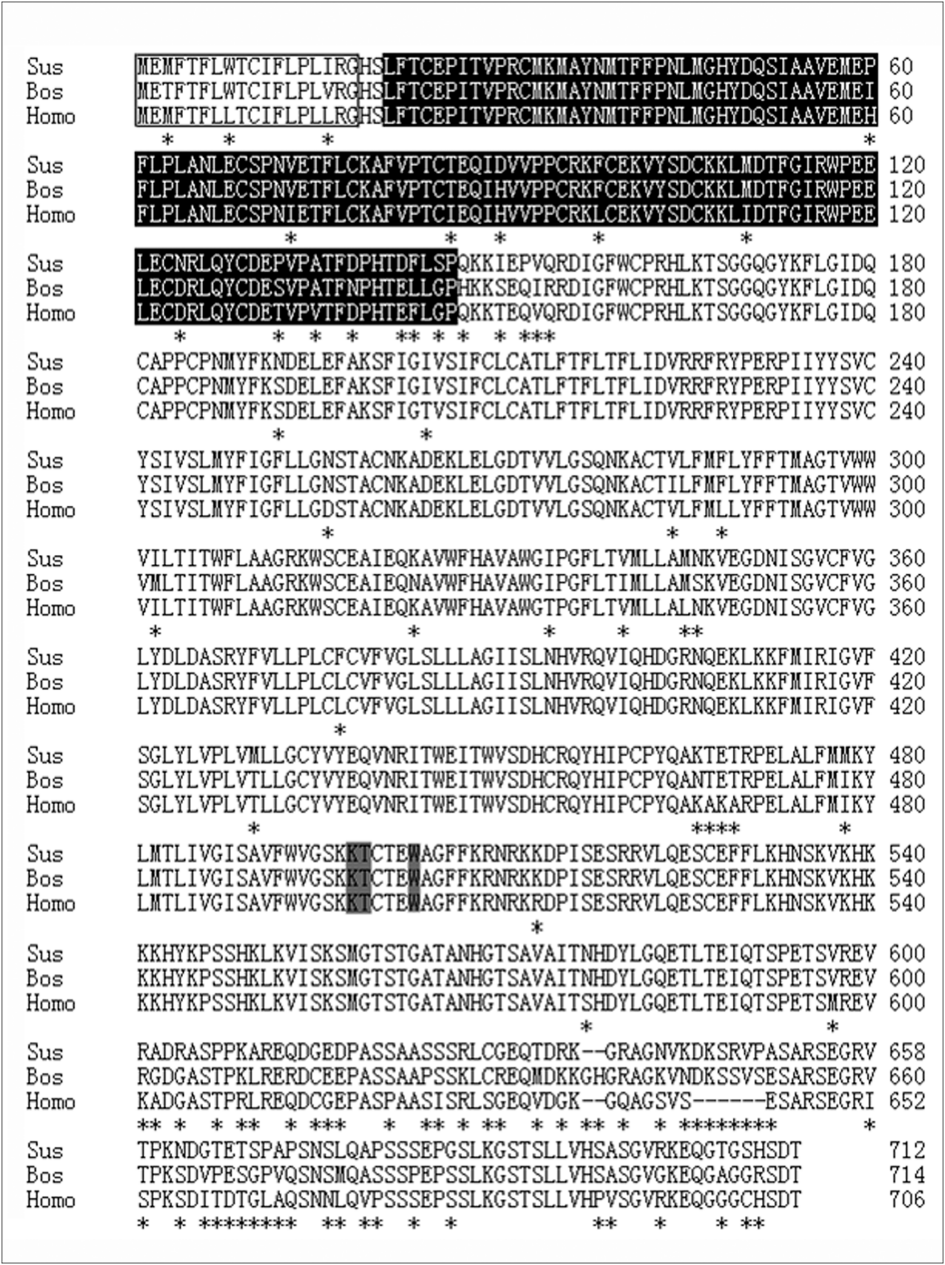
Amino acid sequences alignment of FZD6 from *Sus scrofa*, *Bos Taurus*, and *Homo sapiens*. Differential amino acids are denoted by asterisks. Deletions (-) are introduced in the sequences to maximize the homology. Amino acid numbers are shown on the right of the alignment. The signal peptides are shown in boxes. Cysteine-rich domains (CRD) of the FZD6 receptor are boxed in black. “Lys-Thr-X-X-X-Trp” motifs are shaded in gray.

The *Sfz6* cDNA sequence was obtained from mixed tissue RNA using PCR. The resulting PCR product was 2575 bp. National Center for Biotechnology Information (NCBI) BLAST analysis of the cDNA nucleotide sequence (http://www.ncbi.nlm.nih.gov/BLAST) revealed that the fragment was not homologous to any of the known porcine genes. The sequence was then submitted to the GenBank database (Genbank accession number: KJ808827). Translation of the nucleotide sequence was carried out using the open reading frame (ORF) Finder (http://www.ncbi.nlm.nih.gov/gorf/gorf.html) and an ORF of *Sfz6* cDNA was identified constituting 2139 bp in length and encoding a predicted 712 amino acid (aa) SFz6 protein, which is similar in size to that of other mammalian counterparts: 706 aa (human), 709 aa (murine), and 714 aa (bovine). Hydrophobicity analysis of the aa sequence showed that the hypothetical protein possesses seven transmembrane domains with a cysteine-rich domain at the N-terminal extracellular region, and two cysteine residues in the second and third extracellular loops (Cys 260 and Cys 357). In addition, the putative Sfz6 protein harbors a Lys-Thr-X-X-X-Trp motif at the C-terminal cytoplasmic region (Fig 1).

**Fig 2:**
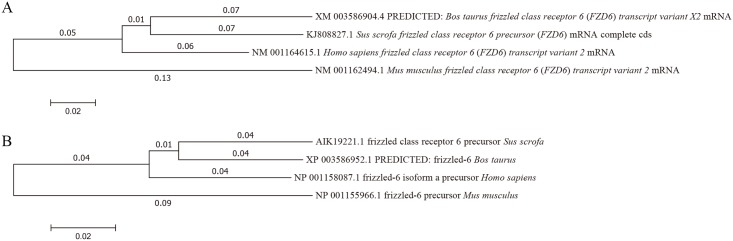
Unrooted phylogenetic tree depicting the evolutionary relationships of FZD6. The unrooted tree was constructed using the neighbor-joining method based on the alignments of the complete gene (A) and aa sequences (B) of known mammalian FZD6 homologues. Scale bar represents substitutions per site; numbers at node points indicate the branch length.

Phylogenetic analyses of the gene and aa sequences were performed, and the resulting neighbor-joining tree showed that *Sfz6* had a closer relationship to *Bfz6* than *Hfz6* and *Mfz6* (Fig 2). Analysis of gene homology indicated that *Sfz6* was 91% similar to *Bfz6*, 90% similar to *Hfz6*, and 82% similar to *Mfz6*. Analysis of protein homology indicated that Sfz6 was 92% similar to Bfz6 protein, 91% similar to Hfz6 protein, and 82% similar to Mfz6 protein. Protein secondary structure prediction was carried out using the Garnier program. The result indicated that the human, pig, cow, and mouse FZD6 proteins share similar secondary structures.

### Tissue expression pattern of *Sfz6* mRNA and protein

**Fig 3:**
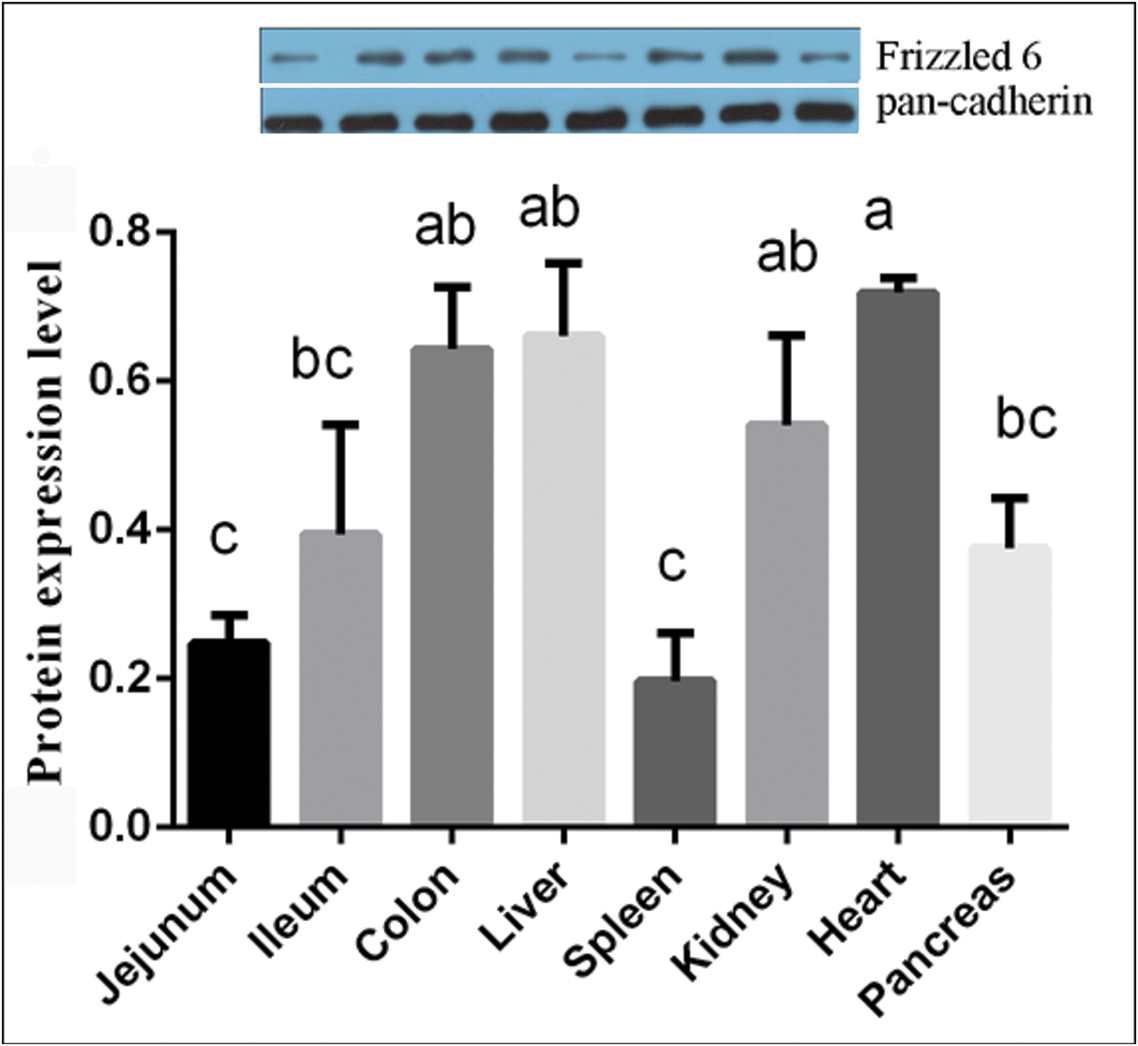
The relative abundance of FZD6 protein expression in the jejunum, ileum, colon, liver, heart, pancreas, spleen, and kidney were analyzed by western blot. Order of Western Blot result has the same as the quantified data. FZD6 protein expression level was analyzed by ratio of FZD6 to pan-cadherin. Values are means ± SEM, n = 6. Values not sharing common letters are significantly different at *P* < 0.05.

The tissue distributions and the relative abundance of *Sfz6* mRNA by qPCR are shown in Table 3. The results show that the pig *FZD6* gene was highly expressed in the kidney (*P*<0.05) and that there was no significant difference of *FZD6* expression in the jejunum, ileum, colon, liver, and spleen. Moreover, there was no significant difference *FZD6* expression in the jejunum, ileum, spleen and heart. In addition, *FZD6* exhibited only weak expression in the pancreas. The expression of FZD6 protein in different tissues is shown in Fig 3. The results showed a high amount of variability with higher expression in the heart, kidney, liver and colon, moderate levels in the ileum and pancreas and lower levels in the jejunum and spleen (Fig 3).

**Table 3:** Expression levels of *Sfz6* mRNA in various tissues.

Tissues (n = 6)	Expression level	SEM
Jejunum	0.039613^bcd^	0.005017
Ileum	0.048043^bcd^	0.006388
Colon	0.095525^b^	0.011816
Liver	0.090091^b^	0.010492
Spleen	0.07101^bc^	0.013726
Kidney	0.276563^a^	0.037673
Heart	0.019745^cd^	0.004177
Pancreas	0.000022^d^	0.000009

Values represent means ± SEM, n = 6. Values not sharing common superscripted letters are significantly different at *P* < 0.05.

### Validation of tissue fractionation and western blot analysis of FZD6

**Fig 4:**
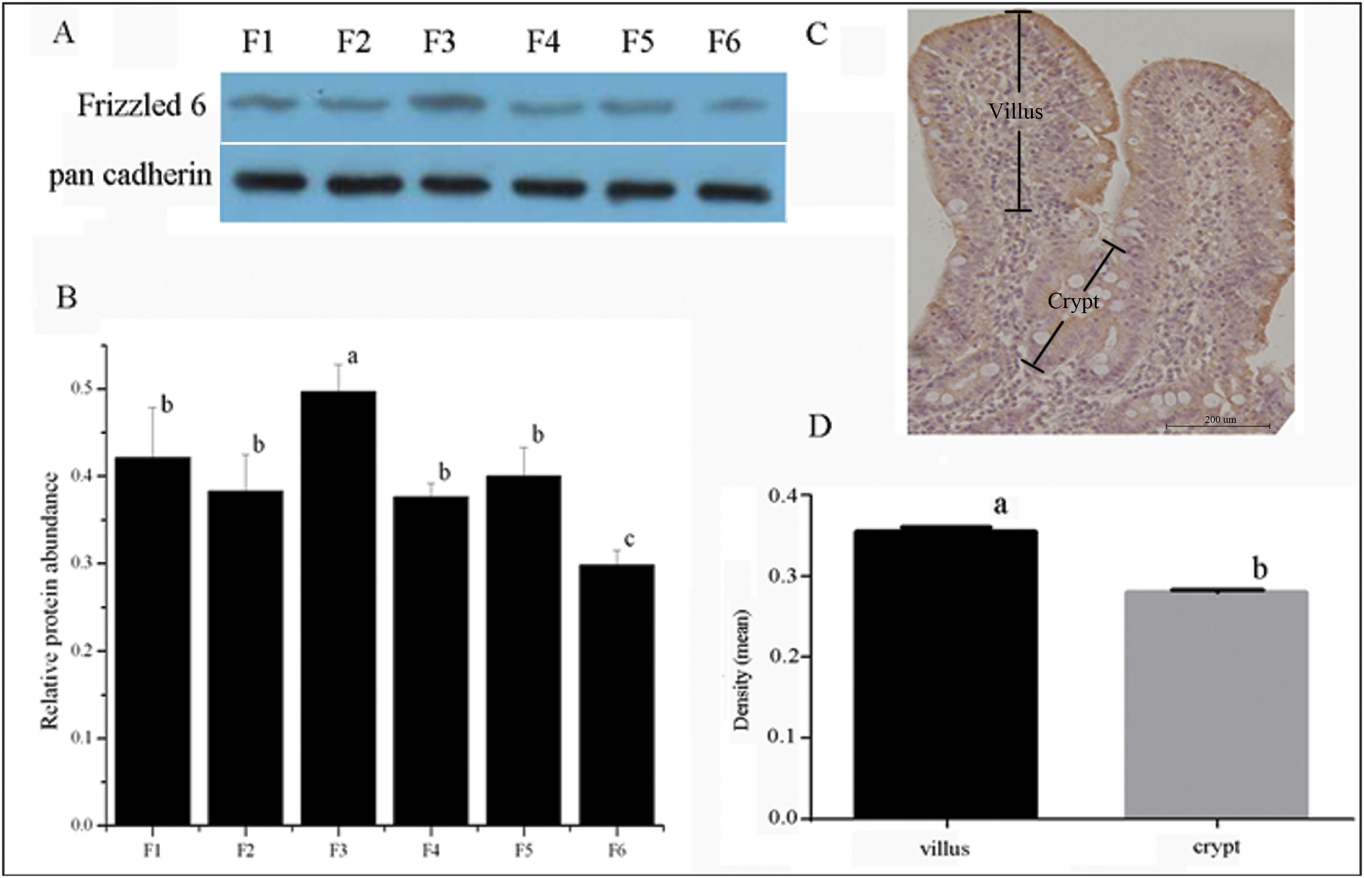
**Protein expression of FZD6 along the jejunal crypt-villus axis was analyzed by western blot (A) and immunohistochemical localization (C).** FZD6 protein expression level was analyzed by ratio of FZD6 to pan-cadherin (B). Quantitative immunohistochemistry analysis of FZD6 staining (D). Values are means ± SEM, n = 6. Values not sharing common letters are significantly different at *P* < 0.05.

We isolated six jejunal epithelial cell fractions along the crypt-villus axis with the method of Fan *et al*. and validated the fractionation efficiency by the measurement of alkaline phosphatase activity, a villus cell marker enzyme. The present study showed that alkaline phosphatase specific activity increased significantly (*P*<0.05) from F6 to F1 (S1 Fig). On the basis of the distribution pattern of the villus cell marker, the 6 cell fractions were grouped as the upper villus (F1-F2), middle villus (F3-F4) and crypt cells (F5-F6). In addition, FZD6 protein showed higher expression in villus epithelial cells than in crypt cells in the jejunum, and F3 exhibited the highest expression of all the cell fractions (Fig 4).

### IHC localization of FZD6 along the jejunal crypt-villus axis

The immunostaining pattern of FZD6 along the jejunal crypt-villus axis is shown in Fig 4C. The IHC image was processed and analyzed with Image-Pro Plus software. The density analysis of FZD6 revealed higher expression in the villus than in the crypt compartment (Fig 4D), which is consistent with the results of western blot analysis of FZD6 in these regions. `

### Effect of *FZD6* knockdown on cell proliferation

**Fig 5:**
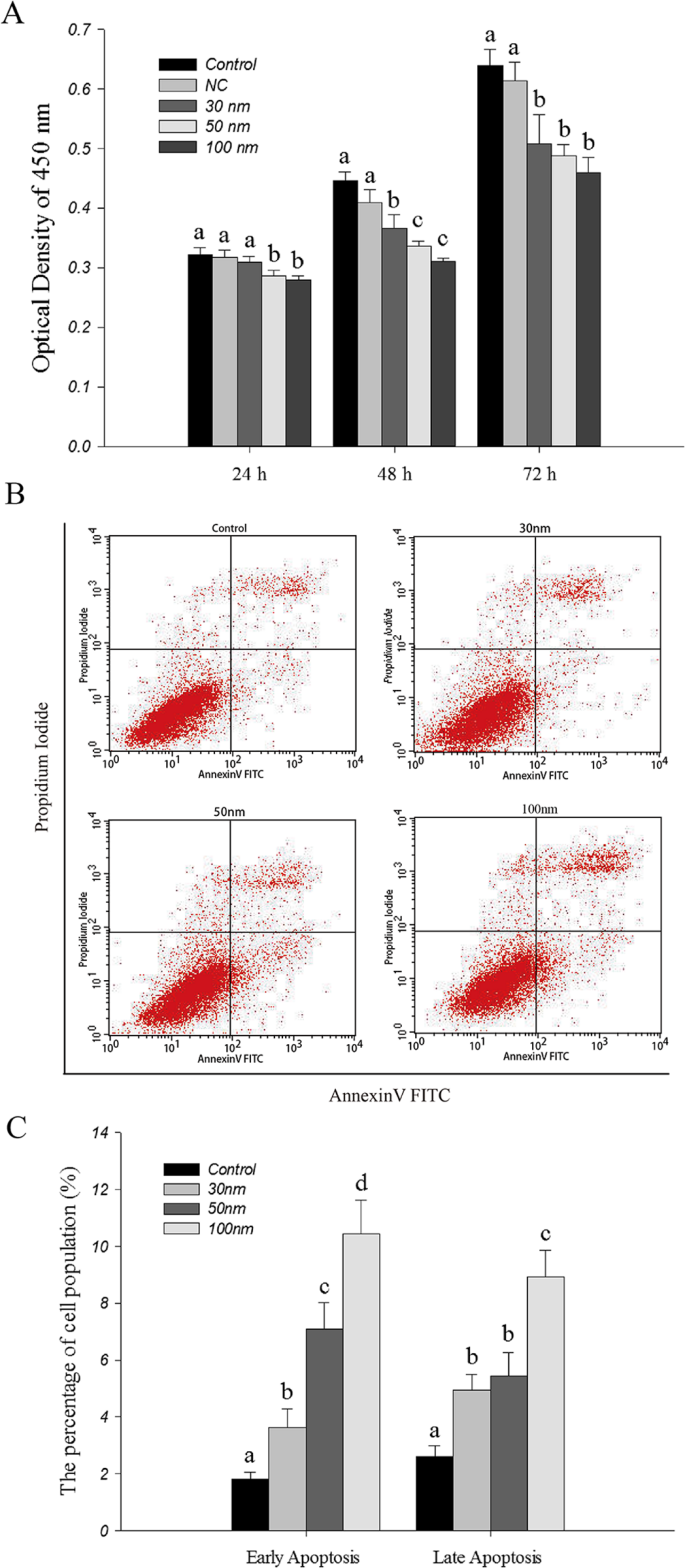
The roles of *FZD6* in cell viability of siRNA treated cells. (A) CCK-8 results of IPEC-J2 cells at different time points. NC = Negative Control; Data are expressed as the mean ±SEM, n = 6. (B) Flow cytometric analysis of Annexin V/PI in IPEC-J2 cells. IPEC-J2 cells were treated with 30nm, 50nm and 100nm *FZD6* siRNA-1 for 48h, respectively. Untreated cells were considered a negative control. (C) Relative apoptosis rate of cells in each group; Data are expressed as the mean ±SEM, n = 3. Values not sharing common letters are significantly different at *P* < 0.05.

To preliminarily evaluate the impact of *FZD6* knockdown in IPEC-J2 cells, qPCR and Western blot was performed to determine the FZD6 expression 48h after transfection. The data showed that the mRNA and protein expression level of FZD6 in *FZD6* siRNA-1 group was significantly decreased compared with siRNA-2 group, siRNA-3 group, the control group and mock group (S2 Fig). Therefore, *FZD6* siRNA-1 was chosen as the interference fragment for the further experiments. *FZD6* siRNA-1 on cell viability of IPEC-J2 cell was measured by CCK-8 assay. The number of cells decreased significantly (*P*<0.05) in *FZD6* siRNA-1 treat group after 48 h and 72h transfection compared with control group (Fig 5A), which indicated that knockdown of *FZD6* expression had an inhibitory effect on the cell proliferation.

### Knockdown of *FZD6* expression induced apoptosis in IPEC-J2 cells

An Annexin V/ PI double staining assay was used to investigate the apoptotic effects of *FZD6* on IPEC-J2 cells. Flow cytometer analysis revealed that *FZD6* siRNA-1 treatment promoted both early and late apoptosis at 48 hours following transfection in the IPEC-J2 cell lines (Fig 5B). Statistical analysis also confirmed that apoptosis was induced in a dose-dependent manner (Fig 5C).The percentage of early apoptotic cells determined by Annexin V-FITC binding cells was significantly increased from 1.82% in untreated cells to 3.62% (30nm), 7.10% (50nm), and 10.44% (100nm) (*P*<0.05), Fig 5C). The percentage of late apoptotic cells (positive for both Annexin V and PI) was significantly increased from 2.62% in untreated cells to 4.95% (30nm), 5.45% (50nm) and 8.94% (100nm) (*P*<0.05, Fig 5C).

### *FZD6* knockdown regulated expression of *RhoA*, *Rac1* and *JNK1* in IPEC-J2 cells

**Fig 6:**
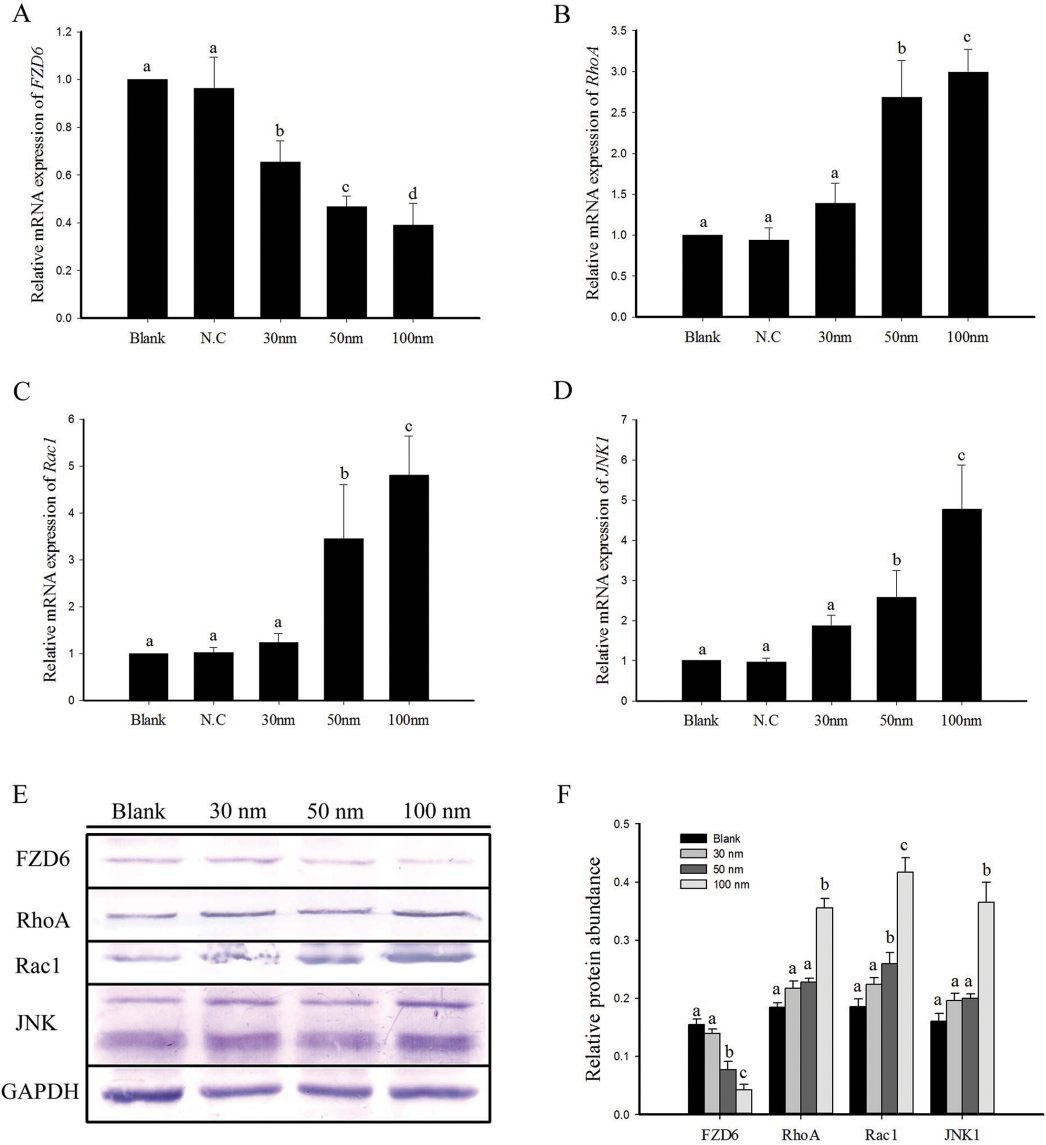
The effect of *FZD6* siRNA on mRNA and protein expressions of FZD6, RhoA, Rac1 and JNK1 in IPEC-J2 cells. After 48 h of *FZD6* siRNA-1 (30nm, 50nm and 100nm) and negative control (100nm) treatment, the mRNA expressions of *FZD6* (A), *RhoA* (B), *Rac1* (C) and *JNK1* (D) in cells were analyzed by qPCR. The mRNA levels were normalized to the expression of GAPDH and plotted as relative mRNA expression. Western blot was performed to identify the protein levels of FZD6, RhoA, Rac1 and JNK1, and GAPDH was used as the control of sample loading (E and F). All results represented as mean ± SEM, n = 3. Values not sharing common letters are significantly different at *P* < 0.05.

The effects of *FZD6* siRNA on the mRNA and protein expression of RhoA, Rac1 and JNK, which are closely related to cell PCP signal pathway, were investigated and the results are presented in Fig 6. The mRNA expression level of *FZD6* significantly decreased (*P*<0.05) compared with control group (Fig 6A). However, the mRNA expression level of *RhoA* (Fig 6B), *Rac1* (Fig 6C) and *JNK1* (Fig 6D) significantly increased (*P*<0.05) in *FZD6* siRNA-1 (50nm and 100nm) treatment compared with control group. The expression of mRNA reached the highest level at *FZD6* siRNA-1 (100nm) treatment group. Western blot analysis showed that the protein expression trends are consistent with mRNA (Fig 6E). The protein expression level of RhoA, Rac1 and JNK significantly increased (*P*<0.05) in *FZD6* siRNA-1 (100nm) treatment compared with control group (Fig 6F).

## Discussion

We have cloned and characterized *Sfz6*, which encodes a seven-transmembrane-spanning receptor (7TMR) with a CRD at the N-terminal extracellular region and the Lys-Thr-X-X-X-Trp motif at the C-terminal cytoplasmic region. The CRD at the N-terminal extracellular region has been implicated in the binding of the receptor cognate ligands, which are proteins of the Wnt family of lipoglycoproteins [4]. The C-terminal cytoplasmic Lys-Thr-X-X-X-Trp motif is thought to mediate Wnt/β-catenin signaling [23]. Taken together, these structural analyses suggest that Sfz6 protein is one of the GPCR protein families. Phylogenetic analysis revealed that FZD6 proteins are highly conserved, structurally and functionally, in mammals. Interestingly however, Sfz6, Hfz6 and Mfz6 all lack the Ser/Thr-X-Val motif, a motif present in some Frizzled family members, and a putative binding site for cytoplasmic proteins containing the Psd-95/disc large/Zo-1 homologous (PDZ) domain [3]. Thus, it is logical to speculate that two divergent signal transduction pathways might exist that functioning through Frizzled subfamilies with or without the C-terminal Ser/The-X-Val motif [3].

*Mfz6* mRNA has been detected by RNase protection assays in the tissues of the adult mice [24]. Comparatively, *Hfz6* has also been detected in various fetal and adult human tissues [3]. In the present study, *Sfz6* mRNA and protein were detected in the jejunum, ileum, colon, liver, heart, pancreas, spleen, and kidney by qPCR and Western blot. Our results showed that Sfz6 is differentially expressed in different tissues. The *Sfz6* mRNA was found to be highly expressed in the kidney, whereas the Sfz6 protein was highly expressed in the heart. In general, it has been well recognized that translational control is important and the most common determinant through which eukaryotic cells regulate gene expression and control target gene protein levels [25]. The absence of mRNA-protein correlationship for the investigated genes suggests that the relation between mRNA and protein is not strictly linear, but has a more intrinsic and complex dependence. The reason maybe that mRNA levels have come down after intervention, or the expression of protein is regulated higher by intracellular activation factor, or the expression of protein is lag [26, 27].

Pig is an excellent model for human nutritional and medical studies, and the pig gastrointestinal tract is very similar to that of human [28]. The intestinal epithelium represents an experimental model for the study of integrated key cellular process such as proliferation and differentiation. This tissue is subjected to a rapid and perpetual self-renewal along the crypt-villus axis [29, 30]. Previous study showed that Wnt-2b, Wnt-4, Wnt-5a, Wnt-5b, FZD4 and FZD6 are also expressed in differentiated epithelial cells of the small intestine and colon [10]. Furthermore, the high expression of *Mfz6* mRNA in mouse crypt cells is higher than that in differentiated cells [10]. In the present study, we identified the expression of the Sfz6 protein in villus differentiated cells (F1-F5) and in crypt cells (F6), and demonstrated that the expression of *Sfz6* was comparatively down-regulated in the latter. The result is different from previous reports in mice or humans, the reason maybe that there exists a cycle interval of differentiation and development of small intestinal epithelial cells. However, there is a need to further clarify the lower expression of *Sfz6* in the villus F1 compared to F3. IHC analysis also revealed that FZD6 protein has higher expression in the villus than in the crypt compartment, which suggests that FZD6 may play a different role in villus cells and crypt cells. Meanwhile, no significant differences were observed between the *Sfz6* mRNA expression levels in the jejunum, ileum, and colon. However, the expression of the Sfz6 protein in the jejunum was lower than that in the ileum and the colon. We speculate that the noted signaling discrepancies might be due to differences in the various villous morphologies and the need for proliferation in the ileum and colon at this time point of intestinal development[31].

The PCP signaling cascade plays a crucial role in the establishment of cell polarity and cell migration [32]. It can activate small GTPases Rac1 and RhoA. The pathway also includes protein kinases rho-associated, coiled-coil-containing protein kinase (ROCK) and JNK that in turn induce cytoskeletal remodeling or elicit a transcriptional response, respectively [33]. Previous studies have revealed that Nitric oxide (NO) inhibits enterocyte migration through activation of RhoA [34]. Recently, it has been shown that the PCP pathway is an important signal cascade for gastrulation via activation of JNK-pathway [35]. Meanwhile, *Rac1* also can mediate apoptosis via *JNK* and plays a key role in proapoptotic pathways in intestinal epithelial cells [36]. Our results show that, after the inhibition of *FZD6* expression, the apoptosis rate of IPEC-J2 cells in siRNA knockdown group was significantly increased compared with the control group (*P*<0.05) and the downstream key genes of PCP pathways (*RhoA*, *Rac1* and *JNK1*) have been significantly up-regulated. Similarly to our finding, previous study showed that there was significant up-regulation of some genes involved in PCP pathway of FZD6^−^ BEAS-2B cell such as *RhoA*, *RAC1*, *DVL1*, and Mitogen-activated protein kinase 9 (*JNK2*) [37]. Therefore, our results indicate that knockdown of *Sfz6* results in an inhibition of cell viability and up-regulated expression of PCP signaling pathway components in IPEC-J2 cell.

We anticipate that further characterization of *Sfz6* will benefit the study of both pig and human intestinal diseases.

## Conclusion

In summary, this is the first report of the cloning and characterization of *Sfz6* gene and necessary analysis of expression level in different tissue. The relatively high level of *FZD6* mRNA was detected at kidney when compared with jejunum, ileum, colon, liver, and spleen, which indicated that the biological function of *FZD6* gene may be regulated by differential expression. IHC analysis of FZD6 revealed that there was higher expression in the villus than in the crypt compartment, which suggests that *FZD6* is related to the cell renewal and migration of the crypt-villus axis. In addition, i*n vitro* studies have showed that knockdown of *FZD6* resulted in decreased IPEC-J2 cell viability and increased PCP signaling pathway components expression.

## Supporting information

S1 Fig Validation of cell fractions isolated from the porcine small intestinal crypt-villus axis. Alkaline phosphatase (ALP) activity was measured at the jejunum along the crypt-villus axis for fractions F1-F6. Values are represented as means ± SEM, n = 8. Values not sharing common letters are significantly different at *P* < 0.05.
(TIF)

S2 Fig Confirmation of *FZD6* siRNA interference efficiency. (A) After 48 h of *FZD6* siRNAs (50nm) and negative control (50nm) treatment, the mRNA expressions of *FZD6* in cells were analyzed by qPCR. (B and C) Western blot was performed to identify the protein levels of FZD6, and GAPDH was used as the control of sample loading. All results represented as mean ±SEM (n = 3). Values not sharing common letters are significantly different at *P* < 0.05.
(TIF)

## References

[1] Nusse R, Brown A, Papkoff J, Scambler P, Shackleford G, McMahon A, et al A new nomenclature for int-1 and related genes: the Wnt gene family. Cell.1991 1 25; 64(2): 231. 10.1016/0092-8674(91)90633-a 1846319

[2] MacDonald BT, Tamai K, He X. Wnt/beta-catenin signaling: components, mechanisms, and diseases. Developmental cell.2009 7; 17(1): 9–26. doi: 10.1016/j.devcel.2009.06.016 19619488 10.1016/j.devcel.2009.06.016 PMC2861485

[3] Tokuhara M, Hirai M, Atomi Y, Terada M, Katoh M. Molecular cloning of human Frizzled-6. Biochemical and biophysical research communications.1998 2 13; 243(2): 622–7. doi: 10.1006/bbrc.1998.8143 9480858 10.1006/bbrc.1998.8143

[4] Schulte G, Bryja V. The Frizzled family of unconventional G-protein-coupled receptors. Trends in pharmacological sciences.2007 10; 28(10): 518–25. doi: 10.1016/j.tips.2007.09.001 17884187 10.1016/j.tips.2007.09.001

[5] Janda CY, Waghray D, Levin AM, Thomas C, Garcia KC. Structural basis of Wnt recognition by Frizzled. Science.2012 7 6; 337(6090): 59–64. doi: 10.1126/science.1222879 22653731 10.1126/science.1222879 PMC3577348

[6] Kilander MB, Dahlstrom J, Schulte G. Assessment of Frizzled 6 membrane mobility by FRAP supports G protein coupling and reveals WNT-Frizzled selectivity. Cellular signalling.2014 9; 26(9): 1943–9. doi: 10.1016/j.cellsig.2014.05.012 24873871 10.1016/j.cellsig.2014.05.012

[7] Kilander MB, Petersen J, Andressen KW, Ganji RS, Levy FO, Schuster J, et al Disheveled regulates precoupling of heterotrimeric G proteins to Frizzled 6. FASEB journal: official publication of the Federation of American Societies for Experimental Biology.2014 5; 28(5): 2293–305. 24500924 10.1096/fj.13-246363

[8] He X, Semenov M, Tamai K, Zeng X. LDL receptor-related proteins 5 and 6 in Wnt/beta-catenin signaling: arrows point the way. Development.2004 4; 131(8): 1663–77. doi: 10.1242/dev.01117 15084453 10.1242/dev.01117

[9] Veeman MT, Axelrod JD, Moon RT. A second canon. Functions and mechanisms of beta-catenin-independent Wnt signaling. Developmental cell.2003 9; 5(3): 367–77. 12967557 10.1016/s1534-5807(03)00266-1

[10] Chang H, Nathans J. Responses of hair follicle-associated structures to loss of planar cell polarity signaling. Proc Natl Acad Sci U S A.2013 3 5; 110(10): E908–17. doi: 10.1073/pnas.1301430110 23431170 10.1073/pnas.1301430110 PMC3593913

[11] Slusarski DC, Corces VG, Moon RT. Interaction of Wnt and a Frizzled homologue triggers G-protein-linked phosphatidylinositol signalling. Nature.1997 11 27; 390(6658): 410–3. doi: 10.1038/37138 9389482 10.1038/37138

[12] Miyakoshi T, Takei M, Kajiya H, Egashira N, Takekoshi S, Teramoto A, et al Expression of Wnt4 in human pituitary adenomas regulates activation of the beta-catenin-independent pathway. Endocrine pathology.2008 Winter; 19(4): 261–73. doi: 10.1007/s12022-008-9048-9 19034702 10.1007/s12022-008-9048-9

[13] Gregorieff A, Pinto D, Begthel H, Destree O, Kielman M, Clevers H. Expression pattern of Wnt signaling components in the adult intestine. Gastroenterology.2005 8; 129(2): 626–38. doi: 10.1016/j.gastro.2005.06.007 16083717 10.1016/j.gastro.2005.06.007

[14] Hughes KR, Sablitzky F, Mahida YR. Expression profiling of Wnt family of genes in normal and inflammatory bowel disease primary human intestinal myofibroblasts and normal human colonic crypt epithelial cells. Inflammatory bowel diseases.2011 1; 17(1): 213–20. doi: 10.1002/ibd.21353 20848536 10.1002/ibd.21353

[15] Yao K, Yin YL, Chu W, Liu Z, Deng D, Li T, et al Dietary arginine supplementation increases mTOR signaling activity in skeletal muscle of neonatal pigs. The Journal of nutrition.2008 5; 138(5): 867–72. 18424593 10.1093/jn/138.5.867

[16] Fan MZ, Stoll B, Jiang R, Burrin DG. Enterocyte digestive enzyme activity along the crypt-villus and longitudinal axes in the neonatal pig small intestine. Journal of animal science.2001 2; 79(2): 371–81. 11219446 10.2527/2001.792371x

[17] Yang HS, Fu DZ, Kong XF, Wang WC, Yang XJ, Nyachoti CM, et al Dietary supplementation with N-carbamylglutamate increases the expression of intestinal amino acid transporters in weaned Huanjiang mini-pig piglets. Journal of animal science.2013 6; 91(6): 2740–8. doi: 10.2527/jas.2012-5795 23478823 10.2527/jas.2012-5795

[18] Geng M, Li T, Kong X, Song X, Chu W, Huang R, et al Reduced expression of intestinal N-acetylglutamate synthase in suckling piglets: a novel molecular mechanism for arginine as a nutritionally essential amino acid for neonates. Amino acids.2011 5; 40(5): 1513–22. doi: 10.1007/s00726-010-0761-6 20931344 10.1007/s00726-010-0761-6

[19] Wang W, Shi C, Zhang J, Gu W, Li T, Gen M, et al Molecular cloning, distribution and ontogenetic expression of the oligopeptide transporter PepT1 mRNA in Tibetan suckling piglets. Amino acids.2009 10; 37(4): 593–601. doi: 10.1007/s00726-008-0178-7 18836683 10.1007/s00726-008-0178-7

[20] Xiong X, Huang S, Zhang H, Li J, Shen J, Xiong J, et al Enrichment and proteomic analysis of plasma membrane from rat dorsal root ganglions. Proteome science.2009 7: 41 doi: 10.1186/1477-5956-7-41 19889238 10.1186/1477-5956-7-41 PMC2780401

[21] Chang J, Chance MR, Nicholas C, Ahmed N, Guilmeau S, Flandez M, et al Proteomic changes during intestinal cell maturation in vivo. Journal of proteomics.2008 12 2; 71(5): 530–46. doi: 10.1016/j.jprot.2008.08.003 18824147 10.1016/j.jprot.2008.08.003 PMC2655360

[22] Feng Z, Guo W, Zhang C, Xu Q, Zhang P, Sun J, et al CCND1 as a predictive biomarker of neoadjuvant chemotherapy in patients with locally advanced head and neck squamous cell carcinoma. PLoS One.2011 6(10): e26399 doi: 10.1371/journal.pone.0026399 22065993 10.1371/journal.pone.0026399 PMC3204964

[23] Umbhauer M, Djiane A, Goisset C, Penzo-Mendez A, Riou JF, Boucaut JC, et al The C-terminal cytoplasmic Lys-thr-X-X-X-Trp motif in frizzled receptors mediates Wnt/beta-catenin signalling. EMBO J.2000 9 15; 19(18): 4944–54. doi: 10.1093/emboj/19.18.4944 10990458 10.1093/emboj/19.18.4944 PMC314225

[24] Wang Y, Macke JP, Abella BS, Andreasson K, Worley P, Gilbert DJ, et al A large family of putative transmembrane receptors homologous to the product of the Drosophila tissue polarity gene frizzled. The Journal of biological chemistry.1996 2 23; 271(8): 4468–76. 8626800 10.1074/jbc.271.8.4468

[25] Sonenberg N, Hinnebusch AG. Regulation of translation initiation in eukaryotes: mechanisms and biological targets. Cell.2009 2 20; 136(4): 731–45. doi: 10.1016/j.cell.2009.01.042 19239892 10.1016/j.cell.2009.01.042 PMC3610329

[26] Gao XF, Li QL, Li HL, Zhang HY, Su JY, Wang B, et al Extracts from Curcuma zedoaria Inhibit Proliferation of Human Breast Cancer Cell MDA-MB-231 In Vitro. Evidence-based complementary and alternative medicine: eCAM.2014 2014: 730678. 24883070 10.1155/2014/730678 PMC4026840

[27] Schulte G. International Union of Basic and Clinical Pharmacology. LXXX. The class Frizzled receptors. Pharmacological reviews.2010 12; 62(4): 632–67. doi: 10.1124/pr.110.002931 21079039 10.1124/pr.110.002931

[28] Guilloteau P, Zabielski R, Hammon HM, Metges CC. Nutritional programming of gastrointestinal tract development. Is the pig a good model for man? Nutrition research reviews.2010 6; 23(1): 4–22. doi: 10.1017/S0954422410000077 20500926 10.1017/S0954422410000077

[29] Mariadason JM, Nicholas, L'Italien KE, Zhuang M, Smartt HJ, Heerdt BG, et al Gene expression profiling of intestinal epithelial cell maturation along the crypt-villus axis. Gastroenterology.2005 4; 128(4): 1081–8. 15825089 10.1053/j.gastro.2005.01.054

[30] Suzuki T, Mochizuki K, Goda T. Localized expression of genes related to carbohydrate and lipid absorption along the crypt-villus axis of rat jejunum. Biochimica et biophysica acta.2009 12; 1790(12): 1624–35. doi: 10.1016/j.bbagen.2009.08.004 19715743 10.1016/j.bbagen.2009.08.004

[31] Leser TD, Molbak L. Better living through microbial action: the benefits of the mammalian gastrointestinal microbiota on the host. Environmental microbiology.2009 9; 11(9): 2194–206. doi: 10.1111/j.1462-2920.2009.01941.x 19737302 10.1111/j.1462-2920.2009.01941.x

[32] Wu J, Roman AC, Carvajal-Gonzalez JM, Mlodzik M. Wg and Wnt4 provide long-range directional input to planar cell polarity orientation in Drosophila. Nat Cell Biol.2013 9; 15(9): 1045–55. doi: 10.1038/ncb2806 23912125 10.1038/ncb2806 PMC3762953

[33] Zallen JA. Planar polarity and tissue morphogenesis. Cell.2007 6 15; 129(6): 1051–63. doi: 10.1016/j.cell.2007.05.050 17574020 10.1016/j.cell.2007.05.050

[34] Cetin S, Leaphart CL, Li J, Ischenko I, Hayman M, Upperman J, et al Nitric oxide inhibits enterocyte migration through activation of RhoA-GTPase in a SHP-2-dependent manner. Am J Physiol Gastrointest Liver Physiol.2007 5; 292(5): G1347–58. doi: 10.1152/ajpgi.00375.2006 17272518 10.1152/ajpgi.00375.2006

[35] Veeman MT, Slusarski DC, Kaykas A, Louie SH, Moon RT. Zebrafish prickle, a modulator of noncanonical Wnt/Fz signaling, regulates gastrulation movements. Current biology: CB.2003 4 15; 13(8): 680–5. 12699626 10.1016/s0960-9822(03)00240-9

[36] Jin S, Ray RM, Johnson LR. Rac1 mediates intestinal epithelial cell apoptosis via JNK. Am J Physiol Gastrointest Liver Physiol.2006 12; 291(6): G1137–47. doi: 10.1152/ajpgi.00031.2006 16798728 10.1152/ajpgi.00031.2006

[37] Piga R, van Dartel D, Bunschoten A, van der Stelt I, Keijer J. Role of Frizzled6 in the molecular mechanism of beta-carotene action in the lung. Toxicology.2014 6 5; 320: 67–73. doi: 10.1016/j.tox.2014.03.002 24657404 10.1016/j.tox.2014.03.002

