# Supplementary figures and images for: Molecular cloning, characterization and expression analysis of Frizzled 6 in the small intestine of pigs (Sus scrofa)
Source: PLoS One. 2017 Jun 14;12(6):e0179421. doi: 10.1371/journal.pone.0179421 (PMC5470702; doi:10.1371/journal.pone.0179421)

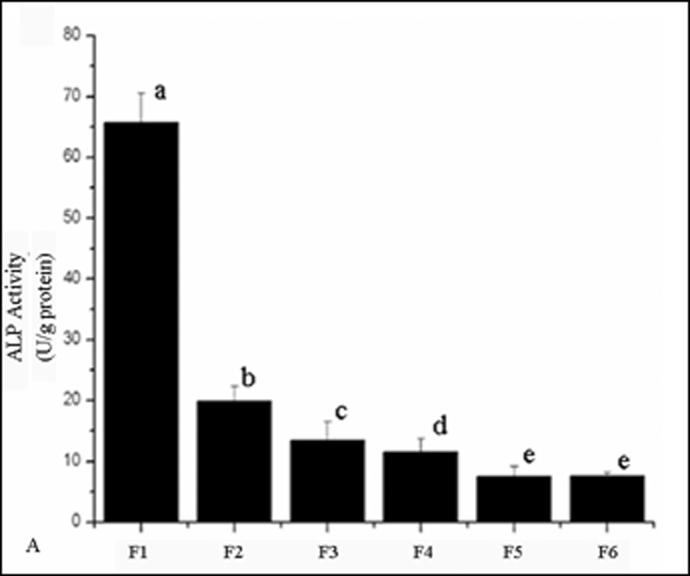

Supplement: S1 Fig — Alkaline phosphatase (ALP) activity was measured at the jejunum along the crypt-villus axis for fractions F1-F6. Values are represented as means ± SEM, n = 8. Values not sharing common letters are significantly different at P < 0.05. (TIF) [file pone.0179421.s001.tif]

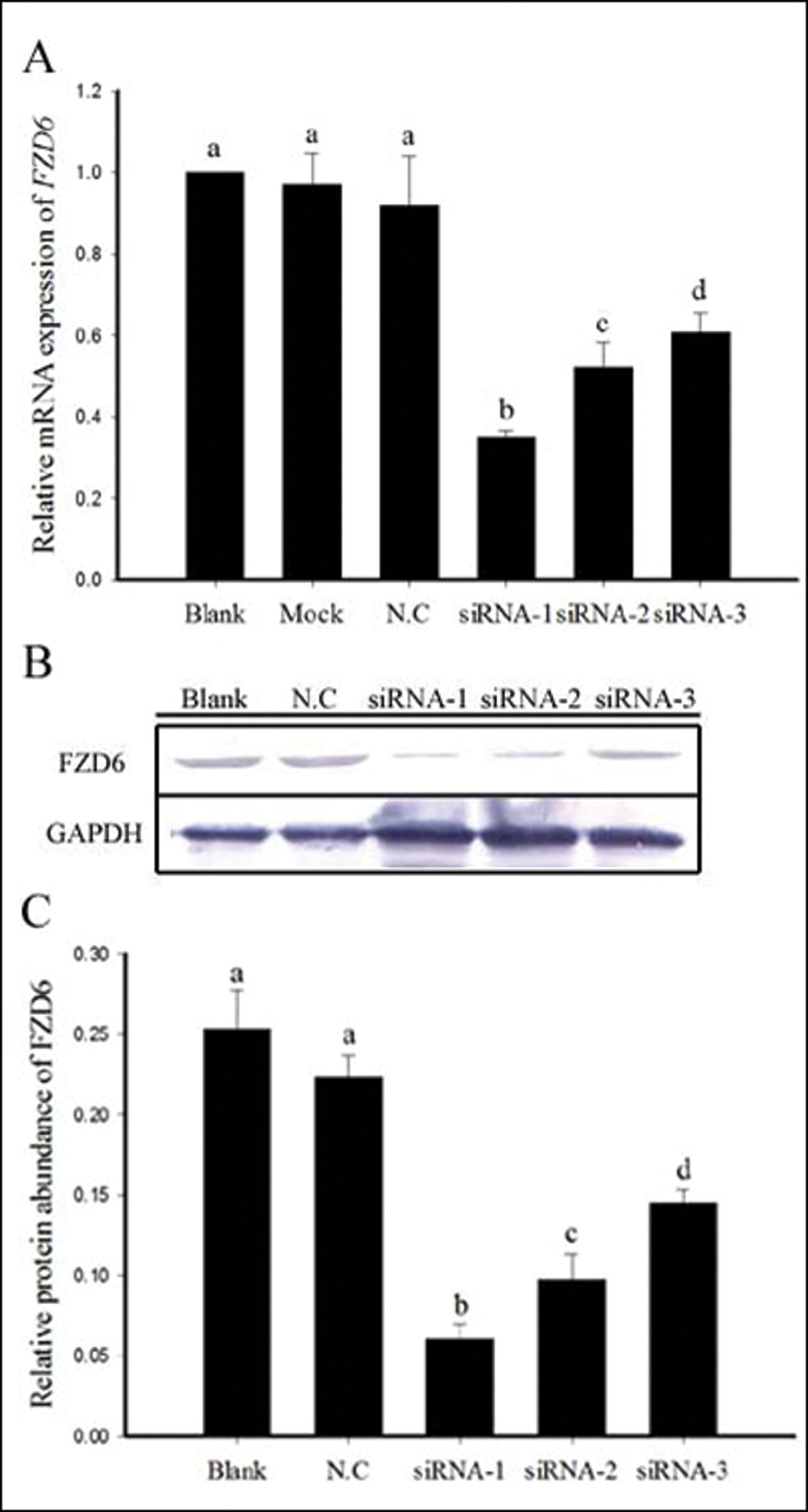

Supplement: S2 Fig — (A) After 48 h of FZD6 siRNAs (50nm) and negative control (50nm) treatment, the mRNA expressions of FZD6 in cells were analyzed by qPCR. (B and C) Western blot was performed to identify the protein levels of FZD6, and GAPDH was used as the control of sample loading. All results represented as mean ±SEM (n = 3). Values not sharing common letters are significantly different at P < 0.05. (TIF) [file pone.0179421.s002.tif]
